# Addition of the FTD Module to the Neuropsychiatric Inventory improves classification of frontotemporal dementia spectrum disorders

**DOI:** 10.1007/s00415-023-11596-3

**Published:** 2023-02-22

**Authors:** Lize C. Jiskoot, Lucy L. Russell, Caroline V. Greaves, Esther van Schaik, Esther van den Berg, Jackie M. Poos, Liset de Boer, Laura Donker Kaat, Harro Seelaar, Yolande A. L. Pijnenburg, John C. van Swieten, Jonathan D. Rohrer

**Affiliations:** 1 Department of Neurology and Alzheimer Center Erasmus MC, Erasmus MC University Medical Center, Room NF-331, Dr. Molenwaterplein 40, 3015 CE Rotterdam, The Netherlands; 2 Dementia Research Centre, Department of Neurodegenerative Disease, University College London Queen Square Institute of Neurology, London, UK; 3 Department of Clinical Genetics, Erasmus Medical Center, Rotterdam, The Netherlands; 4 Department of Neurology, Amsterdam University Medical Centre, Amsterdam, The Netherlands; 5 Dementia Research Centre (DRC), National Hospital for Neurology and Neurosurgery, University College London (UCL), 8-11 Queen Square, Box 16, London, WC1N 3BG UK

**Keywords:** Neuropsychiatric symptoms, Neuropsychiatric inventory, Dementia, FTD, Classification

## Abstract

Most neuropsychiatric symptoms (NPS) common in frontotemporal dementia (FTD) are currently not part of the Neuropsychiatric Inventory (NPI). We piloted an FTD Module that included eight extra items to be used in conjunction with the NPI. Caregivers of patients with behavioural variant FTD (*n* = 49), primary progressive aphasia (PPA; *n* = 52), Alzheimer’s dementia (AD; *n* = 41), psychiatric disorders (*n* = 18), presymptomatic mutation carriers (*n* = 58) and controls (*n* = 58) completed the NPI and FTD Module. We investigated (concurrent and construct) validity, factor structure and internal consistency of the NPI and FTD Module. We performed group comparisons on item prevalence, mean item and total NPI and NPI with FTD Module scores, and multinomial logistic regression to determine its classification abilities. We extracted four components, together explaining 64.1% of the total variance, of which the largest indicated the underlying dimension ‘frontal-behavioural symptoms’. Whilst apathy (original NPI) occurred most frequently in AD, logopenic and non-fluent variant PPA, the most common NPS in behavioural variant FTD and semantic variant PPA were loss of sympathy/empathy and poor response to social/emotional cues (part of FTD Module). Patients with primary psychiatric disorders and behavioural variant FTD showed the most severe behavioural problems on both the NPI as well as the NPI with FTD Module. The NPI with FTD Module correctly classified more FTD patients than the NPI alone. By quantifying common NPS in FTD the NPI with FTD Module has large diagnostic potential. Future studies should investigate whether it can also prove a useful addition to the NPI in therapeutic trials.

**Supplementary Information:**

The online version contains supplementary material available at 10.1007/s00415-023-11596-3.

## Introduction

Frontotemporal dementia (FTD) is the second most common type of young-onset dementia after Alzheimer’s dementia (AD), and is associated with atrophy of the frontal and/or temporal lobes [1–3]. It is commonly sporadic but can be genetic in around a third of people, usually caused by mutations in microtubule-associated protein tau (*MAPT*)*,* chromosome 9 open reading frame 72 (*C9orf72*)*,* and progranulin (*GRN*). The two main clinical manifestations—behavioural variant FTD and primary progressive aphasia (PPA)—are distinguished by their early and predominant symptoms of behavioural or language deterioration respectively [4–6]. Neuropsychiatric symptoms (NPS) are common in behavioural variant FTD, with the majority of patients having behavioural abnormalities as a presenting symptom [7]. Amongst the most frequent are disinhibition, apathy, and altered eating behaviour, whereas mood-related symptoms as anxiety and depression are less frequently reported [5, 8–10]. NPS are less frequent in early-stage PPA but become more prevalent with disease progression [11–13]. NPS accompanying dementia is associated with poor patient and caregiver outcomes such as excess morbidity and mortality increased utilization of health care, and earlier nursing home admittance, as well as significant caregiver distress and burden [14, 15].

The identification and quantification of NPS are important for a timely and accurate diagnosis, the evaluation of disease progression and monitoring of treatment effect and (non-)pharmacological management and the development of effective interventions [15]. The heterogeneity of the FTD spectrum poses major challenges to the development of sensitive measures of disease onset and progression, and clinical endpoint selection for disease-modifying trials [16]. Several scales are available to detect behavioural changes in dementia, such as the Cambridge Behavioural Inventory Revised (CBI-R) [17], Frontal Behavioural Inventory (FBI) [18], and FTD Rating Scale (FRS) [19]. While most of these scales have been developed for or validated in FTD spectrum disorders, they do not solely measure NPS, but also other domains such as (instrumental) activities of daily living, cognition, and mobility. The Neuropsychiatric Inventory (NPI) [20] is one of the most frequently used and investigated questionnaires for NPS in dementia, and has been found to have good differential diagnostic utility [21, 22] as well as good psychometric properties (including content validity, internal consistency, test–retest and interrater reliability) [23]. Differences in NPS between FTD subtypes on the NPI are, however, not well-investigated. A recent study demonstrated higher total NPI scores, as well as more aggressiveness, euphoria, apathy, disinhibition, irritability, aberrant motor behaviours, and appetite disturbances in patients with bvFTD compared to other FTD subtypes (incl. PPA and atypical Parkinsonism), with large overlap among the different FTD subtypes [24]. Moreover, as the NPI was not specifically developed for measuring NPS in the FTD spectrum, certain characteristic symptoms, including those that form part of the clinical diagnostic criteria [5], are not included, such as loss of empathy, less adherence to social norms, and obsessive–compulsive behaviour.

We, therefore, developed an FTD Module of eight additional items characteristic of early-stage FTD, to capture a larger range of NPS. In this study, we piloted the FTD Module in patients with behavioural variant FTD and PPA as well as those with AD and primary psychiatric disorders, aiming to investigate group differences in symptom prevalence and its classification abilities.

## Materials and methods

### Participants and procedure

First, the NPI and next the FTD Module (see below) were administered in written questionnaire format to the caregivers or knowledgeable informants (usually family members) of 280 participants either visiting the outpatient clinic of the Department of Neurology and Alzheimer Center Erasmus Medical Center, Rotterdam, the Netherlands), or participants enrolled in large AD and FTD research cohort studies (Erasmus MC University Medical Center, and Dementia Research Centre, University College London, United Kingdom) between January 2017 and June 2019. We excluded patients with dementia, presymptomatic mutation carriers and controls that met criteria for a psychiatric disorder (Diagnostic and Statistical Manual of Mental Disorders 5th edition; *n* = 3) and patients with primary psychiatric disorders that had cognitive complaints and/or deficits that could be suggestive of a neurodegenerative disease (*n* = 1). Ultimately, we included patients with behavioural variant FTD (*n* = 49; of which 19 carriers of a known pathogenic mutation; *C9orf72* *n* = 11, *MAPT* *n* = 6, *GRN* *n* = 2), PPA (semantic variant PPA *n* = 18, nonfluent variant PPA *n* = 17 (of which 2 carriers of an *GRN* mutation), logopenic variant PPA *n* = 17), AD (*n* = 41), primary psychiatric disorders (*n* = 18, 13 patients with major depressive disorder, 2 with anxiety disorder, 2 with a personality disorder, and 1 with post-traumatic stress disorder), presymptomatic FTD mutation carriers (*n* = 58, *MAPT* *n* = 9, *GRN* *n* = 26, *C9orf72* *n* = 23), and members of FTD families who were mutation negative and therefore acted as cognitively healthy controls (*n* = 58). The clinical diagnoses were made in multidisciplinary consensus meetings, following established diagnostic criteria for behavioural variant FTD [5], PPA [6], AD [25], and psychiatric disorders (Diagnostic and Statistical Manual of Mental Disorders 5th edition). Presymptomatic mutation carriers were deemed asymptomatic according to criteria for either behavioural variant FTD [5] or PPA [6], with a global CDR® plus NACC FTLD score of 0 [26]. The investigators and participants were blinded for the genetic status of presymptomatic mutation carriers and non-carriers, except for those that underwent predictive testing at their own request. 12 presymptomatic mutation carriers and 6 non-carriers were aware of their genetic status. Additionally, we administrated the Mini-Mental State Examination [27], FTD Rating Scale (FRS; [19]), and Cambridge Behavioural Inventory Revised (CBI-R; [17]) to all participants apart from patients with logopenic variant PPA and primary psychiatric disorders. All participants gave written informed consent at the study entry. The study was approved by the Medical and Ethical Review Committee of the Erasmus Medical Centre and University College London Queen Square Institute of Neurology.

### The FTD Module

The FTD Module consists of 8 items (Appendix A), based on a systematic literature review of the behavioural changes described in the FTD spectrum and validated behavioural questionnaires: loss of sympathy/empathy and ritualistic/compulsive behaviour (both part of current diagnostic criteria for bvFTD in addition to disinhibition, apathy and appetite change which are already part of the NPI—[5], poor response to social/emotional cues and inappropriate trusting behaviour (adapted from the Social Impairment Rating Scale (SIRS) —[28] and FBI [18], as well as hyperreligiosity [29, 30], hypersexuality [18, 31], altered sense of humour [18, 32], and altered responsiveness to pain and/or temperature [33]. Caregivers or knowledgeable informants were asked to indicate whether a particular symptom had been present over the last month, and if so, the severity (1 = mild, 2 = moderate, 3 = severe), and frequency (1 = less than weekly, 2 = about once per week, 3 = several times a week, 4 = daily or continuously). Scoring of the FTD Module items was the same as the NPI items. Item total scores were calculated by multiplying severity and frequency scores. Total scores were calculated by adding all item total scores. Addition of the FTD Module score to the maximum total score of 144 of the NPI gave a maximum score of the NPI with FTD Module of 240, with higher scores indicating the presence of more frequent and/or more severe NPS.

### Statistical analysis

We performed statistical analyses using SPSS Statistics 24.0 (IBM Corp., Armonk, NY). The significance level was set at *p* < 0.05 (two-tailed) across all comparisons. We compared continuous demographic data between groups using one-way ANOVA, with Bonferroni post hoc testing. Between-group differences in sex were analysed using chi-square tests. To evaluate the validity and reliability of the NPI with the FTD Module, we investigated the concurrent validity, construct validity (i.e. factor structure) and internal consistency within the total sample. Concurrent validity was determined by Spearman’s rank-order correlation coefficients between the total NPI with FTD Module score and FRS and CBI-R total scores. We assessed construct validity and factor structure by means of a Principal Axis Factoring (PAF) using pro max rotation on the 20 items of the NPI with FTD Module. The Kaiser–Meyer Olkin and Bartlett’s tests of sphericity were used to verify the feasibility of the data for PAF. Only factors accounting for 3% or more of variance and Eigenvalues > 1 were retained. Factor loadings were only considered meaningful when *r* > 0.450, and any item that did not load sufficiently onto a factor was removed [34]. The internal consistency of the total NPI with FTD Module and its underlying components was determined by calculation of Cronbach’s alpha coefficients; values ≥ 0.7 were considered to indicate sufficient internal consistency [35]. We compared item prevalence, and mean item and total NPI and NPI with FTD Module scores by means of Fisher’s exact tests (Bonferroni post hoc testing) and Kruskal–Wallis H Tests (Dunn’s post hoc testing), respectively. We performed multinomial logistic regression analyses on the NPI and NPI with FTD Module total score to determine classification abilities between subgroups. Likelihood ratio chi-square tests were used to establish which items individually significantly contributed to accurate classifications.

## Results

### Demographics

The demographic and clinical characteristics of the behavioural variant FTD, PPA, AD, and primary psychiatric disorder groups as well as the presymptomatic mutation carriers and cognitively healthy controls are presented in Table 1. More presymptomatic mutation carriers and patients with AD were female in comparison with patients with behavioural variant FTD, logopenic variant PPA, and primary psychiatric disorders (all *p* ≤ 0.001). Presymptomatic mutation carriers and controls were significantly younger than patients with behavioural variant FTD, nonfluent variant PPA, logopenic variant PPA and AD (all *p* < 0.001), whereas patients with primary psychiatric disorders were significantly younger than patients with logopenic variant PPA and AD (both *p* < 0.001). Presymptomatic FTD mutation carriers and controls showed significantly less impairment on the MMSE, FAB, FRS and CBI-R in comparison to all other groups (all *p* < 0.001). The highest CDR® plus NACC FTLD sum of box scores was found in patients with behavioural variant FTD, followed by patients with primary psychiatric disorders; the lowest scores were found in presymptomatic mutation carriers and cognitively healthy controls. There were no significant between-group differences in disease duration or years of education.

**Table 1:** Demographic and clinical characteristics of the cohort

Group	bvFTD	svPPA	nfvPPA	lvPPA	AD	PPD	Presymptomatic	Controls	Statistical difference
Sex, female, n (%)	16 (33.3)	7 (38.9)	11 (64.7)	4 (23.5)	29 (70.7)	4 (22.2)	43 (74.1)	36 (62.1)	bvFTD = lvPPA = PPD < AD = presymptomatic
Age, y	62.9 (8.6)	60.5 (9.4)	65.8 (8.5)	71.1 (8.5)	65.5 (7.9)	55.2 (6.9)	50.0 (12.2)	53.0 (12.0)	Presymptomatic = controls < bvFTD = nfvPPA = lvPPA = AD, PPD < lvPPA = AD
Education, y	13.9 (2.3)	14.3 (2.9)	13.2 (2.2)	13.8 (1.9)	12.6 (3.5)	12.6 (3.3)	13.5 (2.7)	13.9 (2.7)	–
Disease duration, m	78 (62)	56 (41)	35 (18)	41 (30)	57 (52)	84 (100)	N/A	N/A	–
MMSE	24.3 (5.4)	25.0 (3.4)	23.7 (5.2)	21.1 (5.2)	18.6 (6.3)	26.6 (3.3)	29.1 (1.6)	29.1 (1.2)	AD = lvPPA < bvFTD = nfvPPA = svPPA = PPD < presymptomatic = controls
FAB	12.2 (4.5)	14.0 (3.6)	11.4 (5.5)	10.3 (4.6)	11.4 (3.4)	15.3 (3.4)	16.9 (1.6)	17.4 (0.9)	lvPPA = AD = nfvPPA < bvFTD = svPPA = PPD < presymptomatic = controls
CDR plus NACC FTLD, SB	8.7 (5.0)	3.8 (4.1)	4.7 (4.3)	3.1 (1.7)	4.5 (4.0)	4.9 (3.9)	0.6 (1.0)	0.8 (1.0)	Controls = presymptomatic = lvPPA = PPD < bvFTD
FRS (%)	46.0 (29.0)	76.5 (12.3)	66.2 (22.2)	71.8 (27.3)	74.0 (8.7)	44.3 (15.0)	96.1 (10.1)	96.2 (9.0)	PPD = bvFTD < svPPA = AD = lvPPA = nfvPPA < presymptomatic = controls
CBI-R^a^	69.5 (31.7)	27.5 (17.7)	48.5 (26.4)	–	49.5 (24.6)	–	4.1 (6.6)	4.5 (7.0)	Controls = presymptomatic < AD = svPPA < bvFTD

Values indicate *n* (%) or mean (*SD*)

Abbreviations: *bvFTD* behavioural variant frontotemporal dementia, *svPPA* semantic variant primary progressive aphasia, *nfvPPA* non-fluent variant primary progressive aphasia, *lvPPA* logopenic variant primary progressive aphasia, *AD* Alzheimer’s Dementia, *PPD* primary psychiatric disorder, *y* years, *m* months, *MMSE* Mini-Mental State Examination, *FAB* Frontal Assessment Battery, *CDR plus NACC FTLD* Clinical Dementia Rating Scale National Alzheimer’s Coordinating Center Frontotemporal Lobar Degeneration, *SB* sum of boxes, *FRS* Frontotemporal dementia Rating Scale, *CBI-R* Cambridge Behavioural Inventory – Revised, *N/A* not applicable

^a^The CBI-R was not administered in patients with lvPPA and patients with primary psychiatric disorders

### Validity and reliability of the NPI with FTD Module

Regarding concurrent validity, the NPI with FTD Module showed a strong negative relationship with the FRS (*n* = 160; *r*_*s*_(158) = − 0.820, *p* < 0.001) and a strong positive correlation with the CBI-R (*n* = 146; *r*_*s*_(144) = 0.854, *p* < 0.001). With respect to construct validity and factor structure, Table 2 shows the results of the PAF with pro max rotation on the 20 items of the NPI with FTD Module. The Kaiser-Meyer Olkin measure was 0.892, indicating great sampling adequacy [35]. Bartlett’s test of sphericity was significant (Chi-Square = 2855.2, *p* < 0.001), indicating that the variables in the correlation matrix were suitable for factor analysis [36]. Based on our criteria and visual inspection of the scree plot, we could extract four components, together explaining 64.1% of the total variance. The first component explained 42.3% of the total variance. Items with the highest loadings were apathy, disinhibition, irritability/lability, aberrant motor behaviour, appetite changes, altered sense of humour, loss of sympathy/empathy, ritualistic/compulsive behaviour, poor response to social/emotional cues, inappropriate trusting behaviour, and altered responsiveness to pain/temperature, indicating the underlying dimension ‘*frontal-behavioural symptoms*’. The second factor explained 10.4% of the total variance. Items with the highest loadings were agitation, depression, apathy, irritability/lability, aberrant motor behaviour, night-time behaviour, and hypersexuality, indicating the underlying dimension ‘*mood symptoms*’. The third component explained 6.0% of the total variance. Items with the highest loadings were delusions, hallucinations, anxiety, and hyperreligiosity, indicating the underlying dimension ‘*psychotic symptoms*’. The fourth and last component only consisted of the item euphoria, explaining 5.4% of the total variance. We, therefore, referred to the last component as ‘*euphoria*’. Overall, the NPI with FTD Module exhibited high internal consistency (*α* = 0.925). The internal consistency could be improved only marginally by removing the items euphoria and hyperreligiosity (*α* = 0.927 and *α* = 0.926, respectively). The ‘*frontal-behavioural symptoms*’ component showed high internal consistency (*α* = 0.918), followed by the ‘*mood symptoms*’ (*α* = 0.775) and ‘*psychotic symptoms*’ (*α* = 0.776) components. In all three components, the removal of any item within that component would lead to lower Cronbach’s alpha coefficients and therefore lower internal consistency.

**Table 2:** Principal Axis Factoring of the NPI with FTD Module

Items	Component coefficients				Communalities
	1 ‘Frontal-behavioural’	2 ‘Mood’	3 ‘Psychotic’	4 ‘Euphoria’	
NPI					
Delusions	0.328	0.239	**0.778**	0.381	0.661
Hallucinations	0.308	0.442	**0.764**	0.110	0.591
Agitation/aggression	**0.643**	**0.788**	0.334	0.411	0.662
Depression/dysphoria	0.361	**0.601**	0.478	0.062	0.429
Anxiety	0.405	**0.647**	**0.713**	0.300	0.618
Euphoria/elation	0.233	0.205	0.157	**0.778**	0.615
Apathy/indifference	**0.717**	**0.614**	0.434	0.243	0.564
Disinhibition	**0.805**	0.317	0.291	0.400	0.673
Irritability/lability	**0.600**	**0.787**	0.396	0.424	0.646
Aberrant motor behaviour	**0.493**	**0.495**	0.286	0.240	0.291
Night-time behaviour	0.426	**0.686**	0.447	0.159	0.493
Appetite/eating	**0.750**	0.101	0.351	0.262	0.616
FTD Module					
Loss of sympathy/empathy	**0.904**	0.281	0.289	0.305	0.818
Ritualistic/compulsive behaviour	**0.694**	0.406	0.342	0.246	0.538
Poor response to social/emotional cues	**0.878**	0.347	0.194	0.373	0.801
Inappropriate trusting behaviour	**0.695**	0.363	0.240	0.188	0.513
Hyperreligiosity	0.233	0.294	**0.655**	0.104	0.436
Hypersexuality	0.351	**0.596**	0.191	0.053	0.196
Altered sense of humour	**0.667**	0.353	0.191	0.306	0.470
Altered responsiveness to pain and/or temperature	**0.622**	0.430	0.293	0.066	0.485
Eigenvalues	8.452	2.078	1.208	1.077	
Variance, %	42.3	10.4	6.0	5.4	

Bold values indicate factor loadings *r* > 0.450

Abbreviations: *NPI* Neuropsychiatric Inventory, *FTD* frontotemporal dementia

### Prevalence

**Fig. 1:**
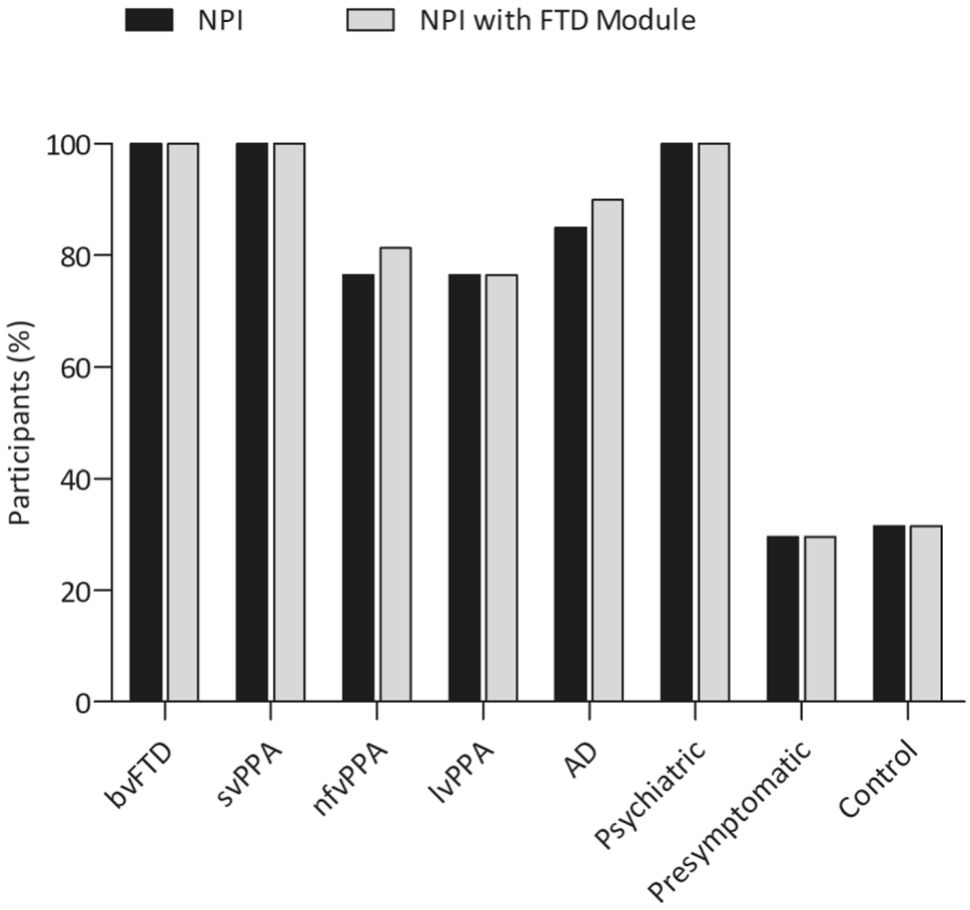
Percentage of participants in each group that showed at least one symptom on the NPI with FTD Module. Abbreviations: *NPI* neuropsychiatric inventory, *FTD* frontotemporal dementia, *bvFTD* behavioural variant frontotemporal dementia, *svPPA* semantic variant primary progressive aphasia, *nfvPPA* non-fluent variant primary progressive aphasia, *lvPPA* logopenic variant primary progressive aphasia, *AD* Alzheimer’s dementia

**Fig. 2:**
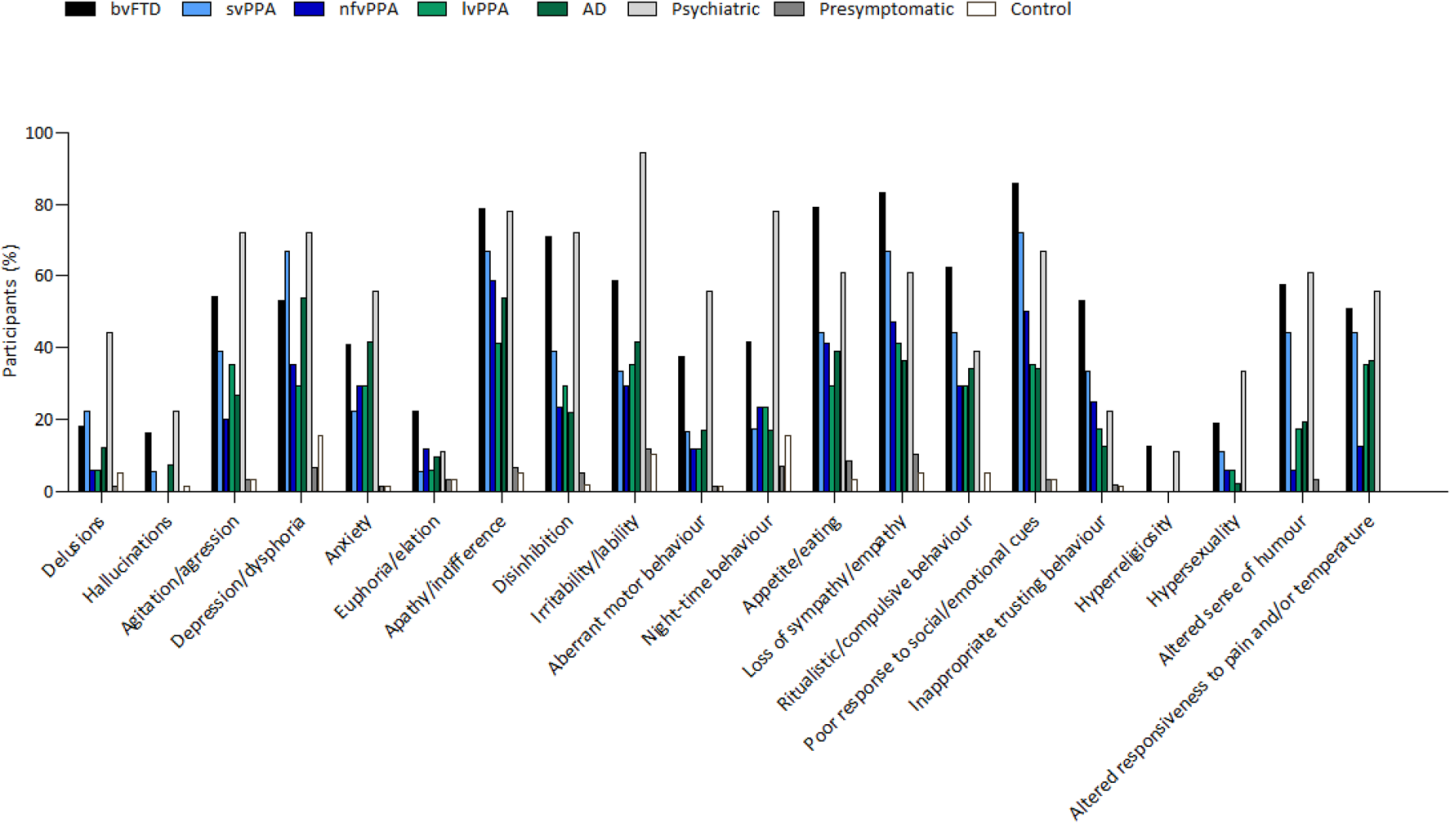
Percentage of patients in each group that showed each individual NPI with FTD Module item. Abbreviations: *NPI* neuropsychiatric inventory, *FTD* frontotemporal dementia, *bvFTD* behavioural variant frontotemporal dementia, *svPPA* semantic variant primary progressive aphasia, *nfvPPA* non-fluent variant primary progressive aphasia, *lvPPA* logopenic variant primary progressive aphasia, *AD* Alzheimer’s dementia

Figure 1 shows the percentage of participants in each group that presented with at least one symptom on the NPI and NPI with the FTD Module. All patients with behavioural variant FTD, semantic variant PPA and primary psychiatric disorders showed disturbances on at least one item of the NPI and NPI with FTD Module. However, NPS were not present in all patients with nonfluent variant PPA, logopenic variant PPA and AD, and were relatively uncommon in presymptomatic FTD mutation carriers and controls. Figure 2 shows the prevalence of all NPI and FTD Module items in each group (also shown in Supplementary Table S1). Loss of empathy and a poor response to social/emotional cues were the most common symptoms in patients with behavioural variant FTD and semantic variant PPA, while apathy was the most frequent symptom in patients with nonfluent variant PPA, logopenic variant PPA and AD. In patients with primary psychiatric disorders, irritability was the most frequently reported symptom. Both the NPI and NPI with FTD Module showed that overall behavioural abnormalities were significantly more common in patients with bvFTD in comparison to presymptomatic FTD mutation carriers and controls (all *p* < 0.001). More specifically, symptoms of agitation, depression, anxiety, apathy, disinhibition, irritability, aberrant motor behaviour, and changes in appetite and eating were significantly more common in patients with behavioural variant FTD in comparison to presymptomatic FTD mutation carriers and controls (all *p* < 0.001). Patients with bvFTD patients further showed a significantly higher prevalence on all items of the FTD Module in comparison to presymptomatic FTD mutation carriers and controls (all *p* ≤ 0.001), with the exception of hyperreligiosity (both *p* = 0.007). When compared with other groups, patients with behavioural variant FTD additionally showed more prevalent NPS in comparison to patients with nonfluent variant PPA, logopenic variant PPA and AD. Patients with behavioural variant FTD more often showed disinhibition and alterations in humour in comparison to patients with nonfluent variant PPA (*p* = 0.001 and *p* < 0.001, respectively), whilst appetite and eating changes and a poor response to social/emotional cues were significantly more prevalent in behavioural variant FTD in comparison to patients with logopenic variant PPA (both* p* < 0.001). Patients with behavioural variant FTD also more frequently presented with disinhibition, appetite and eating changes, alterations in humour, loss of empathy, reductions in the response to social cues and inappropriate trusting behaviour in comparison to patients with AD (all* p* < 0.001). Lastly, behavioural variant FTD patients did not show any significant differences with regard to the prevalence of any NPS in comparison to semantic variant PPA and primary psychiatric disorders. The total prevalence scores of the NPI and NPI with FTD Module demonstrated that behavioural abnormalities were significantly more prevalent in patients with semantic variant PPA in comparison to the presymptomatic FTD mutation carriers and controls (all *p* < 0.001). Patients with semantic variant PPA did not show any significant differences with regard to the prevalence of specific NPS in comparison to any other patient groups, except for the items of irritability and night-time behaviour, which were significantly less common than in patients with primary psychiatric disorders (both *p* < 0.001). Presymptomatic FTD mutation carriers did not show any significant differences with regard to the presence of overall or specific behavioural problems in comparison to controls, as shown by similar scores on the total NPI with the FTD Module and all individual items of the NPI and FTD Module.

### Mean scores

**Fig. 3:**
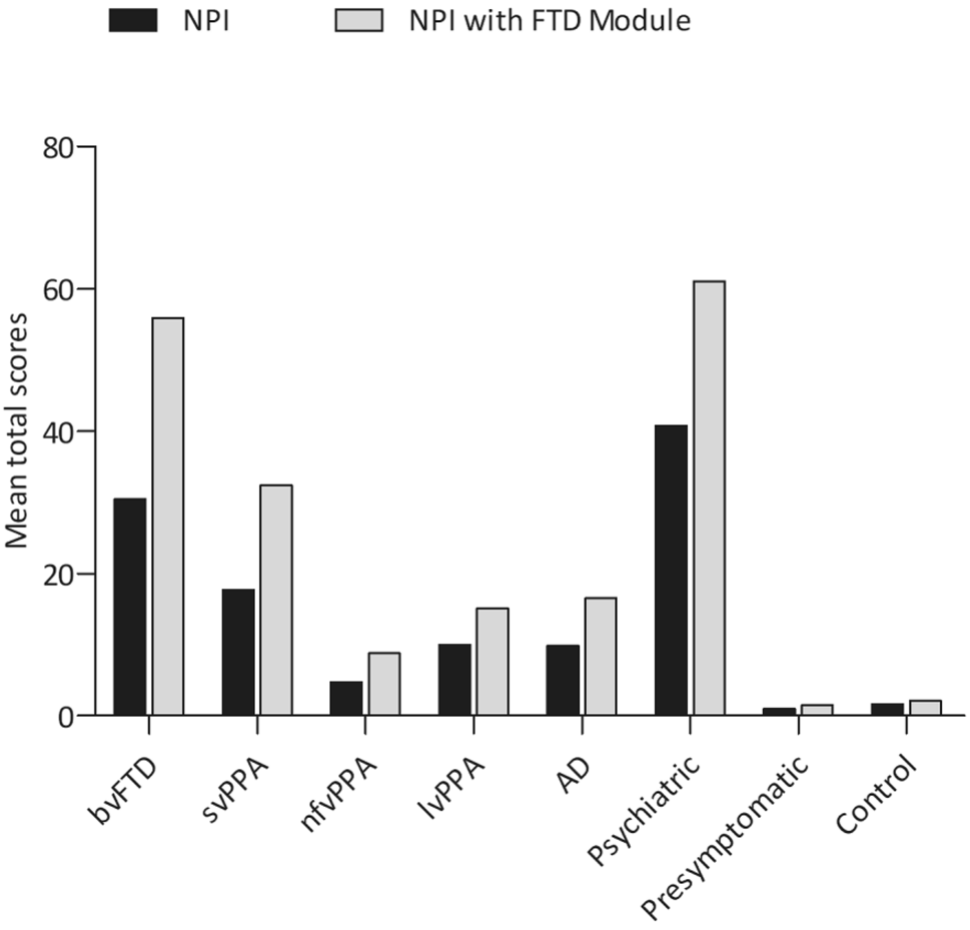
NPI with FTD Module scores of each group. Abbreviations: *NPI* neuropsychiatric inventory, *FTD* frontotemporal dementia, *bvFTD* behavioural variant frontotemporal dementia, *svPPA* semantic variant primary progressive aphasia, *nfvPPA* non-fluent variant primary progressive aphasia, *lvPPA* logopenic variant primary progressive aphasia, *AD* Alzheimer’s dementia

**Fig. 4:**
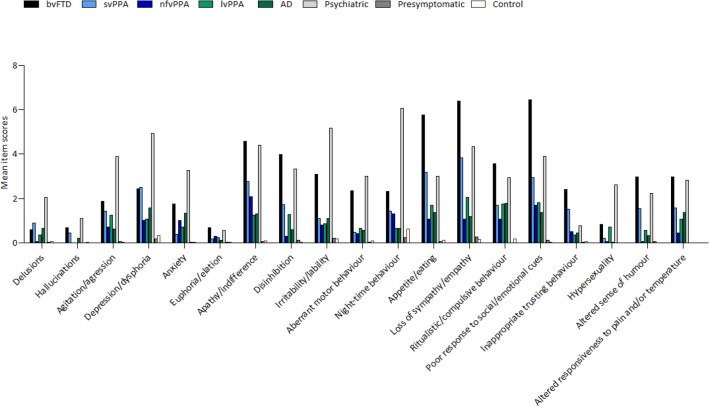
NPI with FTD Module individual item scores for each group. Abbreviations: *NPI* neuropsychiatric inventory, *FTD* frontotemporal dementia, *bvFTD* behavioural variant frontotemporal dementia, *svPPA* semantic variant primary progressive aphasia, *nfvPPA* non-fluent variant primary progressive aphasia, *lvPPA* logopenic variant primary progressive aphasia, *AD* Alzheimer’s dementia

Mean total and individual item (severity*frequency) NPI with FTD Module scores of each group are presented in Figs. 3 and 4 (and Supplementary Table S2). Patients with primary psychiatric disorders showed the most severe behavioural problems on both the NPI as well as the NPI with FTD Module, followed by patients with behavioural variant FTD. Of the PPA variants, patients with semantic variant PPA showed the most severe behavioural problems, and patients with nonfluent variant PPA showed the least severe. Patients with logopenic variant PPA, on the other hand, had total mean scores in between the other two PPA variants, and showed similar levels of behavioural dysfunction as those with AD. Presymptomatic FTD mutation carriers and controls only showed mild behavioural dysfunction on both the NPI and NPI with FTD Module. In general, the items of the NPI with FTD Module could separate patients with behavioural variant FTD from presymptomatic FTD mutation carriers and controls, as patients with bvFTD scored significantly higher on all items of the NPI with FTD Module (all *p* ≤ 0.001), except for delusions (*p* = 0.008 and *p* = 0.034, respectively) and hyperreligiosity (both *p* = 0.003). Patients with behavioural variant FTD demonstrated significantly worse disinhibition, appetite and eating changes, changes in humour, loss of empathy, poor response to social/emotional cues and alterations in the responsiveness to pain and/or temperature in comparison to patients with nonfluent variant PPA (all *p* ≤ 0.001). Moreover, patients with behavioural variant FTD showed significantly worse apathy, disinhibition, appetite and eating changes, changes in humour, loss of empathy, poor response to social/emotional cues and inappropriate trusting behaviour in comparison to patients with logopenic variant PPA and AD (all *p* ≤ 0.001). Patients with behavioural variant FTD did not show significant differences in mean scores on any item of the NPI with FTD Module in comparison to patients with semantic variant PPA. Moreover, patients with behavioural variant FTD only showed significantly milder night-time behaviour (*p* < 0.001) than primary psychiatric disorders, but no other significant differences between the two groups were seen.

The NPI and NPI with FTD Module both demonstrated worse behavioural problems for patients with semantic variant PPA in comparison to presymptomatic FTD mutation carriers and controls (all *p* < 0.001), but not in comparison to any other group. Patients with semantic variant PPA presented with significantly more severe depression, apathy, changes in humour, loss of empathy, poor response to social/emotional cues and alterations in the responsiveness to pain and/or temperature in comparison to presymptomatic FTD mutation carriers and controls, as well as more severe ritualistic/compulsive behaviour in comparison to presymptomatic FTD mutation carriers only (all *p* ≤ 0.001). Besides significantly milder symptoms of irritability, aberrant motor behaviour and night-time behaviour in comparison to patients with primary psychiatric disorders (all *p* < 0.001), patients with semantic variant PPA did not significantly differ from other groups with regard to the severity of specific NPS. Presymptomatic FTD mutation carriers did not show significant differences with regard to the severity of overall or specific behavioural problems in comparison to controls, as represented by comparable mean scores on the total NPI and NPI with FTD Module, and all individual items of the NPI and FTD Module.

### Classification abilities of the NPI and NPI with FTD Module

Table 3 presents the percentages of correctly classified and misclassified participants in each group by the NPI and NPI with FTD Module, respectively. The classification abilities of the NPI and NPI with FTD Module can be found in Table 4. The NPI model was statistically significant (*χ*^2^(84) = 306.79, *p* < 0.001) and explained 72.1% of the total variance (Nagelkerke *R*^2^ = 0.721). Overall, the NPI correctly classified a total of 47.4% of all participants, with the best classifications for patients with behavioural variant FTD (74.4% correct) and presymptomatic FTD mutation carriers (83.0% correct), and worst classifications for patients with nonfluent variant PPA and logopenic variant PPA (both 0% correct). Predictors significantly contributing to the classification of the NPI were the items delusions (*χ*^2^(7) = 18.89, *p* < 0.001), agitation (*χ*^2^(7) = 20.08, *p* = 0.005), anxiety (*χ*^2^(7) = 26.10, *p* < 0.001), apathy (*χ*^2^(7) = 30.33, *p* < 0.001) and appetite (*χ*^2^(7) = 21.81, *p* = 0.003). The NPI with FTD Module model was also statistically significant (*χ*^2^(140) = 446.32, *p* < 0.001, respectively) and explained 86.0% of the total variance (Nagelkerke *R*^2^ = 0.860). The NPI with FTD Module correctly classified a total of 58.8% of all participants. The NPI with FTD Module best classified presymptomatic FTD mutation carriers (86.8% correct), patients with behavioural variant FTD (82.9% correct) and patients with primary psychiatric disorders (83.3% correct), and showed worst classifications for the controls (23.2% correct) and patients with nonfluent variant PPA (22.2% correct). Predictors significantly contributing to the classification of the NPI with FTD Module were the NPI items delusions (*χ*^2^(7) = 23.94, *p* = 0.001), agitation (*χ*^2^(7) = 22.72, *p* = 0.002), anxiety (*χ*^2^(7) = 20.72, *p* = 0.004), apathy (*χ*^2^(7) = 32.42, *p* < 0.001), irritability/lability (*χ*^2^(7) = 21.75, *p* = 0.003), night-time behaviour (*χ*^2^(7) = 31.67, *p* < 0.001) and appetite (*χ*^2^(7) = 15.00, *p* = 0.036), as well as the FTD Module items poor response to social/emotional cues (*χ*^2^(7) = 29.71, *p* < 0.001), hypersexuality (*χ*^2^(7) = 14.27, *p* = 0.047), and responsiveness to pain and/or temperature (*χ*^2^(7) = 29.00, *p* < 0.001). The NPI with FTD Module was able to classify between patients with nonfluent variant PPA and controls and between patients with semantic variant PPA and patients with logopenic variant PPA, where the NPI could not (Table 4).

**Table 3:** Classification of the groups with the NPI and the NPI Module

Group	bvFTD	svPPA	nfvPPA	lvPPA	AD	PPD	Presymptomatic	Control
Classification NPI (%)								
bvFTD	**74.4**	2.3	0	0	7.0	7.0	7.0	2.3
svPPA	13.3	**33.3**	0	6.7	13.3	0	20.0	13.3
nfvPPA	18.2	0	**0**	0	9.1	0	54.5	18.2
lvPPA	11.8	5.9	0	**0**	41.2	0	35.3	5.9
AD	13.2	2.6	0	0	**42.1**	2.6	26.3	13.2
Psychiatric	22.2	0	0	0	16.7	**55.6**	0	5.6
Presymptomatic	0	0	0	1.9	1.9	0	**83.0**	13.2
Control	0	0	0	0	1.8	1.8	75.0	**21.4**
Classification NPI with FTD Module (%)								
bvFTD	**82.9**	0	0	0	7.3	2.4	2.4	4.9
svPPA	7.1	**42.9**	0	7.1	28.6	7.1	0	7.1
nfvPPA	11.1	0	**22.2**	0	22.2	0	44.4	0
lvPPA	0	0	0	**42.8**	21.4	0	28.6	7.1
AD	10.5	2.6	2.6	7.9	**55.3**	2.6	18.4	0
Psychiatric	5.6	0	0	5.6	5.6	**83.3**	0	0
Presymptomatic	0	0	0	0	0	0	**86.8**	13.2
Control	0	0	0	1.8	1.8	0	73.2	**23.2**

Values indicate the percentage of participants of each group that was correctly classified (bold values), and percentage of participants of each group that was misclassified as belonging to another group

Abbreviations: *bvFTD* behavioural variant frontotemporal dementia, *svPPA* semantic variant primary progressive aphasia, *nfvPPA* non-fluent variant primary progressive aphasia, *lvPPA* logopenic variant primary progressive aphasia, *AD* Alzheimer’s Dementia, *PPD* primary psychiatric disorder, *NPI* Neuropsychiatric Inventory

**Table 4:** Classification abilities of the NPI and NPI with FTD Module per subgroup

	AUC	95% CI	*p* value	Optimal cut-off	Sensitivity (%)	Specificity (%)
bvFTD vs. controls						
NPI	0.97	0.93–1.00	< 0.001	5.0	92.7	91.1
NPI with FTD Module	0.97	0.95–1.00	< 0.001	8.5	92.7	92.9
bvFTD vs. svPPA						
NPI	0.71	0.53–0.88	0.021	20.5	70.7	78.6
NPI with FTD Module	0.70	0.54–0.87	0.024	38.5	70.7	71.4
bvFTD vs. AD						
NPI	0.82	0.73–0.91	< 0.001	18.5	73.2	84.2
NPI with FTD Module	0.84	0.75–0.93	< 0.001	39.0	70.7	89.5
bvFTD vs. PPD						
NPI	0.40	0.57–0.77	0.220	–	–	–
NPI with FTD Module	0.50	0.65–0.69	0.799	–	–	–
bvFTD vs. presymptomatic mc						
NPI	0.98	0.96–1.00	< 0.001	7.5	90.2	96.2
NPI with FTD Module	0.98	0.96–1.00	< 0.001	11.5	87.8	98.1
svPPA vs. controls						
NPI	0.92	0.85–0.98	< 0.001	0.5	100	69.6
NPI with FTD Module	0.94	0.88–1.00	< 0.001	3.5	85.7	87.5
svPPA vs. nfvPPA						
NPI	0.78	0.58–0.98	0.025	10.0	57.1	88.9
NPI with FTD Module	0.79	0.60–0.97	0.023	12.5	71.4	77.8
svPPA vs. lvPPA						
NPI	0.68	0.47–0.88	0.108	–	–	–
NPI with FTD Module	0.74	0.55–0.93	0.035	12.0	71.4	71.4
nfvPPA vs. controls						
NPI	0.69	0.49–0.89	0.070	–	–	–
NPI with FTD Module	0.73	0.53–0.93	0.028	4.5	55.6	89.3
nfvPPA vs. lvPPA						
NPI	0.38	0.62–0.86	0.345	–	–	–
NPI with FTD Module	0.44	0.69–0.80	0.637	–	–	–
lvPPA vs. controls						
NPI	0.76	0.60–0.92	0.003	3.0	64.3	89.3
NPI with FTD Module	0.77	0.61–0.93	0.002	4.5	64.3	89.3
lvPPA vs. AD						
NPI	0.46	0.63–0.72	0.621	–	–	–
NPI with FTD Module	0.42	0.59–0.76	0.353	–	–	–
AD vs. controls						
NPI	0.82	0.73–0.91	< 0.001	0.5	84.2	69.6
NPI with FTD Module	0.86	0.78–0.94	< 0.001	2.5	76.3	85.7
AD vs. PPD						
NPI	0.89	0.80–0.97	< 0.001	13.5	76.3	88.9
NPI with FTD Module	0.86	0.76–0.96	< 0.001	16.0	68.4	88.9
PPD vs. controls						
NPI	0.99	0.96–1.00	< 0.001	5.0	100	91.1
NPI with FTD Module	0.99	0.97–1.00	< 0.001	9.0	100	92.2
Presymptomatic mc vs. controls						
NPI	0.49	0.60–0.62	0.825	–	–	–
NPI with FTD Module	0.49	0.59–0.62	0.783	–	–	–

Abbreviations: *NPI* Neuropsychiatric Inventory, *bvFTD* behavioural variant frontotemporal dementia, *svPPA* semantic variant primary progressive aphasia, *nfvPPA* non-fluent variant primary progressive aphasia, *lvPPA* logopenic variant primary progressive aphasia, *AD* Alzheimer’s Dementia, *PPD* primary psychiatric disorder, *mc* mutation carrier

## Discussion

We describe an FTD Module with eight symptoms characteristic of FTD not currently included in the NPI. Using this FTD Module in addition to the NPI allowed quantification of key NPS that were highly prevalent in behavioural variant FTD and semantic variant PPA, such as loss of sympathy/empathy and poor response to social/emotional cues. Furthermore, the addition of the FTD Module improved the classification of the FTD spectrum disorders compared with the NPI alone. Overall there was high concurrent validity, construct validity and internal consistency of the NPI with the FTD Module.

We extracted four components, together explaining 64.1% of the total variance. We called the first and largest component, explaining 42.3% of the total variance, ‘frontal-behavioural’, as it consisted of NPS characteristic for FTD spectrum disorders, such as apathy, disinhibition, appetite changes, loss of sympathy/empathy, ritualistic/compulsive behaviour, and poor response to social/emotional cues. Prior research into the factor structure of the NPI in patients with dementia demonstrated both comparable as well as different underlying components. Similar to our results, both Aalten et al. [37] and Kang et al. [38] identified a mood/affect and a psychosis cluster in their data, however, they also found a hyperactivity syndrome that was not found in our factor analysis. Differences between prior studies and ours are most likely due to the addition of the FTD Module to the NPI and the inclusion of different dementia diagnoses, as ours focused on FTD spectrum disorders, and Aalten et al. [37] and Kang et al. [38] mostly included patients with AD and vascular dementia, and not FTD spectrum disorders. Our factor analysis also identified a ‘psychotic’ component, with high loadings from the NPS delusions, hallucinations, and hyperreligiosity. This is an interesting finding, as psychotic symptoms are thought to be relatively rare in FTD [39]. An explanation for a psychotic component in our data could be that we have included presymptomatic and symptomatic *C9orf72* repeat expansion carriers, in whom psychotic symptoms (e.g., delusions, hallucinations, mania) can be the leading clinical presentation [40]. As evidenced by previous studies, hyperreligiosity tends to be quite specific for FTD spectrum disorders (e.g., [29, 30, 41]). Although well-described in temporal lobe epilepsy and primary psychiatric disorders (e.g., schizophrenia, psychosis), patients with other forms of dementia than FTD do usually not exhibit hyperreligiosity. Studies into hyperreligiosity have suggested atrophy of the right temporal lobe as its anatomical underpinning (e.g., [29, 30, 41]), potentially explaining the relatively high prevalence in FTD spectrum disorders.

Most previous studies using the NPI in dementia have been focused on comparing patients with behavioural variant FTD and patients with AD (e.g., [42–45]). Similar to our study, higher total NPI scores were found before in patients with behavioural variant FTD than in patients with AD [42–45]. With respect to the individual items, also comparable results were found, with patients with behavioural variant FTD having higher scores on disinhibition [42–45] and appetite and eating changes [45]. Agitation, euphoria an aberrant motor behaviour, as found in these previous studies, were not significantly different in our study. A potential explanation can be found in the more atypical presentations of AD that are seen in academic outpatient memory clinics such as ours, including patients with the behavioural/dysexecutive variant that clinically shows much overlap with behavioural variant FTD [46]. It is interesting that in our study patients with behavioural variant FTD had more changes in the FTD Module items of humour, loss of empathy/sympathy, poor response to social/emotional cues and inappropriate trusting behaviour in comparison to patients with AD, as it would suggest that these items could be useful in the differential diagnosis between the two disease entities. Studies of the NPI within the FTD spectrum are scarce, with only one study thus far comparing different clinical subtypes [24]. The authors found significantly higher NPI total scores in patients with behavioural variant FTD than in patients with corticobasal syndrome, nonfluent variant PPA, progressive supranuclear palsy, and semantic variant PPA. In contrast to the study by Yiannopoulou et al. [24], all our patients with semantic variant PPA had at least one NPS, had high NPI (with FTD Module) total and item scores, and did not show significant differences in NPI (with FTD Module) total and item scores in comparison to patients with behavioural variant FTD. In contrast, some of the patients with logopenic variant PPA and nonfluent variant PPA had no NPS, and overall displayed mild NPS. This corroborates the findings from previous studies (e.g. [47]) that demonstrated that semantic variant PPA is associated with similar behavioural and NPS as behavioural variant FTD, and patients demonstrate more behavioural dysfunction than the other PPA variants. No increase in NPS was seen in the presymptomatic FTD mutation carriers. Previous studies into NPS in the presymptomatic phase of FTD have shown higher apathy [48, 49], agitation, disinhibition, elation and irritability [49] in comparison to non-carriers. The fact that we did not find presymptomatic changes may well be due to the relatively small numbers in our sample, and a wide variety of ages in this group, including participants who were likely to be some time of developing symptoms. Rohrer et al. [50] indeed found NPS five years before estimated symptom onset, suggesting that behavioural and NPS appear relatively late in the presymptomatic stage of FTD.

The model with the NPI with FTD Module overall led to better classification of patients. More specifically, the NPI items delusions, agitation, anxiety, apathy, irritability, night-time behaviour and appetite, as well as the FTD Module items poor response to social/emotional cues, hypersexuality, and responsiveness to pain and/or temperature were significant contributors, leading to the largest improvement in patients with behavioural variant FTD, presymptomatic FTD mutation carriers and patients with primary psychiatric disorders. Overall classification going from 47.4 to 58.8% with the inclusion of the FTD Module is an important improvement, but also shows that there is likely significant overlap in FTD spectrum disorders to have a ‘perfect’ classification between the different conditions. In comparison to a previous study [43], the classification of patients with bvFTD with the NPI is better (74% vs. 54%), while the classification of patients with AD is worse (42% vs. 87%) in our sample. A possible explanation can be sought in the use of different statistical analyses (NPI total score vs. symptom clusters) and the use of different diagnostic criteria ([5] vs. [51]), potentially leading to higher diagnostic accuracy in our bvFTD patients. Again, the likely inclusion of atypical presentations of AD (behavioural/dysexecutive variant) could have lowered our diagnostic accuracy of AD patients. Poor classification of patients with nfvPPA, lvPPA and controls is most likely due to the fact that not all of these patients have NPS and total NPI scores are generally low. Therefore it is more interesting that ~ 87% of presymptomatic FTD mutation carriers was correctly classified by the NPI with FTD Module. This suggests that the NPI with FTD Module provides items sensitive and specific to the earliest stages of the disease process, which is a promising finding given the upcoming disease-modifying trials in presymptomatic FTD.

A key strength of this study is our large total sample of patients, covering different clinical subtypes in both the symptomatic as well as presymptomatic phase of FTD. Using the original NPI and adding the eight items still makes the NPI with FTD Module a fast, easy, and flexible instrument to be used in clinical practice and future clinical trials. It could be argued that a study of this type entails a certain degree of circularity, in that the FTD Module is comprised of NPS common in behavioural variant FTD and was used for its clinical diagnosis in this study, inevitably improving the differential diagnosis among this FTD subtype vs. other dementia types in comparison to the original NPI. Moreover, as most clinical diagnoses were not pathologically confirmed, there is a small possibility that patients were misdiagnosed (e.g., patients with behavioural/dysexecutive AD as behavioural variant FTD, and behavioural variant FTD patients with prominent memory deficits as AD), with lower classification accuracy between dementia subtypes as a result. A limitation of the study is the relatively small sample sizes per patient subgroup, and replication using larger samples is warranted. Future studies using the FTD Module in FTD mutation carriers within the last five to ten years prior to estimated symptom onset will be helpful in understanding whether more subtle presymptomatic NPS can be picked up with this questionnaire. Moreover, longitudinal studies and subanalyses in the different FTD gene mutations will shed light on the development of NPS with approaching disease onset and mutation-specific NPS profiles in genetic FTD. Repeated administration of the NPI with FTD Module in a short-time-interval will allow the monitoring of within-person variability—and investigation of intra-rater reliability—in NPS in FTD spectrum disorders, as a recent study showed strong fluctuations in NPI scores in a memory clinic population [52]. Moreover, test–retest and inter-rater reliability of the FTD Module should be investigated in future studies. Finally, long-term assessments of NPS using the NPI with FTD Module and other behavioural questionnaires (e.g., CBI-R, FBI, FRS) are needed to assess the evolution of NPS and their correlations during the course of the disease [53].

In summary, the FTD Module offers the ability to detect and quantify a set of NPS that are not currently included in the NPI. Whilst previous attempts have been made to make completely new questionnaires, here we wanted to make use of the extensive knowledge about the NPI and make an FTD Module that might be used alongside the well-validated NPI rather than starting again. We hope that future studies can investigate its use further, and in particular, whether it might be used with the NPI as a clinical outcome measure in therapeutic trials for FTD.

## Supplementary Information

Below is the link to the electronic supplementary material.

Supplementary file1 (DOCX 57 KB)

## Data Availability

Anonymized data not published within this article will be made available upon reasonable request from any qualified investigator.
