# Supplementary material for: Addition of the FTD Module to the Neuropsychiatric Inventory improves classification of frontotemporal dementia spectrum disorders
Source: J Neurol. 2023 Feb 22;270(5):2674–87. doi: 10.1007/s00415-023-11596-3 (PMC10129920; doi:10.1007/s00415-023-11596-3)
Supplement: Supplementary file 1 — Supplementary file1 (DOCX 57 KB) [file 415_2023_11596_MOESM1_ESM.docx]

# Appendix A

# FTD Module

- We would like to ask you a number of questions about changes in the patient’s behaviour that you may have noticed. Your view is important as it will help us in our assessment. Please read each question carefully. For each question, indicate the *frequency* of the behaviour, followed by the level of *severity*.
- **All questions apply to the patient’s behaviour over the past one month.**

|  | **Does this symptom occur?** | **How often?** | | | | **How severe?** | | |
| --- | --- | --- | --- | --- | --- | --- | --- | --- |
|  |  | Less than weekly | About once per week | Several times a week | Daily or continuously | Mild | Moderate | Severe |
|  |  | 1 | 2 | 3 | 4 | 1 | 2 | 3 |
| **Loss of sympathy/empathy** | | | | | | | | |
| Does the patient seem to respond less to other people’s needs or feelings? Is there reduced personal warmth? Do they seem to lack empathy or sympathy for others? | Yes ☐  (then answer how often and how severe)  No ☐  (then proceed to next question) | Less than weekly | About once per week | Several times a week | Daily or continuously | Loss of sympathy or empathy is notable but is only mildly different to patient’s usual behaviour | Loss of sympathy or empathy is very evident and occurs in a substantial number of interactions with people | Loss of sympathy or empathy is very evident and occurs in the majority of interactions with other people |
| **Ritualistic/compulsive behaviour** | | | | | | | | |
| Does the patient engage in compulsive behaviours such as continually checking, counting or cleaning things? Does the patient have fixed rituals or routines that they must perform? | Yes ☐  (then answer how often and how severe)  No ☐  (then proceed to next question) | Less than weekly | About once per week | Several times a week | Daily or continuously | Ritualistic or compulsive behaviours are notably present but are not disturbing | Ritualistic or compulsive behaviours are very evident but may be able to be overcome by the caregiver | Ritualistic or compulsive behaviours are very evident and usually do not respond to any intervention by the caregiver |
| **Poor response to social/emotional cues** | | | | | | | | |
| Does the patient seem to be worse at interacting socially with others e.g. is there less eye contact or do they stand closer than normal to others? Are they insensitive to how a normal conversation flows? Do they seem to have difficulty reading other’s emotions? | Yes ☐  (then answer how often and how severe)  No ☐  (then proceed to next question) | Less than weekly | About once per week | Several times a week | Daily or continuously | Pays noticeably less attention to social cues, or sometimes responds awkwardly or unexpectedly to social cues | Pays much less attention to social cues, or often responds awkwardly or unexpectedly to social cues | Pays almost no attention to social cues, or very often responds awkwardly or unexpectedly to social cues |
| **Inappropriate trusting behaviour** | | | | | | | | |
| Does the patient seem more gullible e.g. have they been taken advantage of? Are they less cautious than before? Have they performed any acts of poor judgment e.g. fallen for scams or purchased something extravagant they wouldn’t have done before? | Yes ☐  (then answer how often and how severe)  No ☐  (then proceed to next question) | Less than weekly | About once per week | Several times a week | Daily or continuously | Has displayed a few clear but minor acts of poor judgment of other people | Has displayed multiple minor acts or a few major acts of poor judgment of other people resulting in adverse consequences | Has displayed recurrent, in at least some cases severe, acts of poor judgment of other people resulting in adverse consequences |
| **Hyperreligiosity** | | | | | | | | |
| Does the patient seem to have developed increased or unusual religious experiences or feelings compared to previously? | Yes ☐  (then answer how often and how severe)  No ☐  (then proceed to next question) | Less than weekly | About once per week | Several times a week | Daily or continuously | Increased or unusual religious experiences are present but seem harmless and produce little distress | Increased or unusual religious experiences may be distressing and disruptive | Increased or unusual religious experiences are very disruptive and are a major source of behavioural disruption |
| **Hypersexuality** | | | | | | | | |
| Has the patient started to engage in unusual or excessive sexual behaviour? Do they have increased libido compared to before? | Yes ☐  (then answer how often and how severe)  No ☐  (then proceed to next question) | Less than weekly | About once per week | Several times a week | Daily or continuously | Sexual behaviour is disruptive but can be managed by redirection or reassurance | Sexual behaviour is disruptive and difficult to redirect or control | Sexual behaviour is very disruptive and a major source of difficulty |
| **Altered sense of humour** | | | | | | | | |
| Has the patient developed a change in sense of humour, such as finding things funny which they didn’t before or finding things funny that others don’t? | Yes ☐  (then answer how often and how severe)  No ☐  (then proceed to next question) | Less than weekly | About once per week | Several times a week | Daily or continuously | Mild but definite change in sense of humour. May find things funny which others don’t | Sense of humour completely different from previously (e.g. may prefer more physical or slapstick humour) | Complete loss of sense of humour |
| **Altered responsiveness to pain and/or temperature** | | | | | | | | |
| Has the way that the patient seems to perceive pain or temperature changed? Do they seem to have a reduced or heightened response to pain? Do they seem to have changed in how they tolerate heat or cold (e.g. wearing less clothes when it’s cold, or more clothes when it’s hot)? | Yes ☐  (then answer how often and how severe)  No ☐  (then proceed to next question) | Less than weekly | About once per week | Several times a week | Daily or continuously | Mild change in responsiveness to pain or temperature – no limitation on daily activities | Moderate change in responsiveness to pain or temperature – some limitation on daily activities | Severe change in responsiveness to pain or temperature – limits most daily activities |

# Supplementary Table S1. Prevalence of individual NPI with FTD Module items in each group.

|  | bvFTD | svPPA | nfvPPA | lvPPA | AD | PPD | Presymptomatic | Controls | *p* | Statistical difference |
| --- | --- | --- | --- | --- | --- | --- | --- | --- | --- | --- |
| NPI | | | | | | | | | | |
| Delusions | 9 (18.4) | 4 (22.2) | 1 (5.9) | 1 (5.9) | 5 (12.2) | 8 (44.4) | 1 (1.7) | 3 (5.2) | <0.001 | con = pre = AD = nfvPPA = lvPPA < PDD |
| Hallucinations | 8 (16.3) | 1 (5.6) | 0 (0) | 0 (0) | 3 (7.3) | 4 (22.2) | 0 (0) | 1 (1.7) | 0.001 | con = pre < PDD = bvFTD |
| Agitation/Aggression | 26 (54.2) | 7 (38.9) | 3 (20.0) | 6 (35.3) | 11 (26.8) | 13 (72.2) | 2 (3.4) | 2 (3.4) | <0.001 | con = pre = AD = svPPA = lvPPA < bvFTD = PDD |
| Depression/Dysphoria | 26 (53.1) | 12 (66.7) | 6 (35.3) | 5 (29.4) | 22 (53.7) | 13 (72.2) | 4 (6.9) | 9 (15.5) | <0.001 | pre = con < AD = bvFTD = svPPA = PDD |
| Anxiety | 20 (40.8) | 4 (22.2) | 5 (29.4) | 5 (29.4) | 17 (41.5) | 10 (55.6) | 1 (1.7) | 1 (1.7) | <0.001 | pre = con < AD = bvFTD = PDD |
| Euphoria/Elation | 11 (22.4) | 1 (5.6) | 2 (11.8) | 1 (5.9) | 4 (9.8) | 2 (11.1) | 2 (3.4) | 2 (3.4) | 0.032 | pre = con < bvFTD |
| Apathy/Indifference | 37 (78.7) | 12 (66.7) | 10 (58.8) | 7 (41.2) | 22 (53.7) | 14 (77.8) | 4 (6.9) | 3 (5.2) | <0.001 | pre = con = lvPPA < bvFTD = AD |
| Disinhibition | 34 (70.8) | 7 (38.9) | 4 (23.5) | 5 (29.4) | 9 (22.0) | 13 (72.2) | 3 (5.2) | 1 (1.8) | <0.001 | con = pre = AD = svPPA =  nfvPPA = lvPPA < bvFTD = PDD |
| Irritability/Lability | 28 (58.6) | 6 (33.3) | 5 (29.4) | 6 (35.3) | 17 (41.5) | 17 (94.4) | 7 (12.1) | 6 (10.3) | <0.001 | con = pre < AD = bvFTD = svPPA  = nfvPPA = lvPPA < PDD |
| Aberrant motor behaviour | 18 (37.5) | 3 (16.7) | 2 (11.8) | 2 (11.8) | 7 (17.1) | 10 (55.6) | 1 (1.7) | 1 (1.7) | <0.001 | con = pre < bvFTD = AD = nfvPPA  = svPPA = lvPPA < PDD |
| Night-time behaviour | 20 (41.7) | 3 (17.6) | 4 (23.5) | 4 (23.5) | 7 (17.1) | 14 (77.8) | 4 (7.0) | 9 (15.5) | <0.001 | con = pre < bvFTD = AD = nfvPPA  = svPPA = lvPPA < PDD |
| Appetite/Eating | 38 (79.2) | 8 (44.4) | 7 (41.2) | 5 (29.4) | 16 (39.0) | 11 (61.1) | 5 (8.6) | 2 (3.5) | <0.001 | con = pre < AD = svPPA = nfvPPA = lvPPA < bvFTD |
| FTD Module | | | | | | | | | | |
| Loss of sympathy/empathy | 40 (83.3) | 12 (66.7) | 8 (47.1) | 7 (41.2) | 15 (36.6) | 11 (61.1) | 6 (10.3) | 3 (5.3) | <0.001 | con = pre < AD = nfvPPA = lvPPA  < bvFTD = svPPA = PDD |
| Ritualistic/compulsive behaviour | 30 (62.5) | 8 (44.4) | 5 (29.4) | 5 (29.4) | 14 (34.1) | 7 (38.9) | 0 (0) | 3 (5.3) | <0.001 | con = pre < AD < bvFTD = svPPA = PDD |
| Poor response to social/emotional cues | 42 (85.7) | 13 (72.2) | 8 (50.0) | 6 (35.3) | 14 (34.1) | 12 (66.7) | 2 (3.4) | 2 (3.4) | <0.001 | con = pre < AD = lvPPA = nfvPPA  < bvFTD = svPPA = PDD |
| Inappropriate trusting behaviour | 26 (53.1) | 6 (33.3) | 4 (25.0) | 3 (17.6) | 5 (12.5) | 4 (22.5) | 1 (1.8) | 1 (1.7) | <0.001 | con = pre = AD = PDD < bvFTD = svPPA |
| Hyperreligiosity | 6 (12.5) | 0 (0) | 0 (0) | 0 (0) | 0 (0) | 2 (11.1) | 0 (0) | 0 (0) | 0.001 | con = pre = AD < bvFTD |
| Hypersexuality | 9 (19.1) | 2 (11.1) | 1 (5.9) | 1 (5.9) | 1 (2.4) | 6 (33.3) | 0 (0) | 0 (0) | <0.001 | con = pre = AD = nfvPPA = lvPPA < bvFTD = PDD |
| Altered sense of humour | 27 (57.4) | 8 (44.4) | 1 (5.9) | 3 (17.6) | 8 (19.5) | 11 (61.1) | 2 (3.4) | 0 (0) | <0.001 | con = pre = AD = nfvPPA = lvPPA  < bvFTD = PDD = svPPA |
| Altered responsiveness to pain and/or temperature | 25 (51.0) | 8 (44.4) | 2 (12.5) | 6 (35.3) | 15 (36.6) | 10 (55.6) | 0 (0) | 0 (0) | <0.001 | con = pre < AD = nfvPPA < PDD = svPPA  = lvPPA = bvFTD = svPPA |
| Total NPI - 1 or more NPS on the NPI | 49 (100) | 18 (100) | 13 (76.5) | 13 (76.5) | 34 (85.0) | 18 (100) | 16 (29.6) | 18 (31.6) | <0.001 | con = pre < AD = bvFTD = nfvPPA  = svPPA = lvPPA = PDD |
| Total NPI with FTD Module – 1 or more NPS on the NPI with FTD Module | 49 (100) | 18 (100) | 13 (81.3) | 13 (76.5) | 36 (90.0) | 18 (100) | 16 (29.6) | 18 (31.6) | <0.001 | con = pre < AD = bvFTD = nfvPPA  = svPPA = lvPPA = PDD |
| Values indicate *n* (%). Abbreviations: bvFTD, behavioural variant frontotemporal dementia; svPPA, semantic variant primary progressive aphasia; nfvPPA, non-fluent variant primary progressive aphasia; lvPPA, logopenic variant primary progressive aphasia; AD, Alzheimer’s Disease; NPI, Neuropsychiatric Inventory; FTD, frontotemporal dementia; pre, presymptomatic mutation carrier; con, controls. | | | | | | | | | | |

Supplementary Table S2. Mean scores on the individual items of the NPI with FTD Module.

| **Item** | **bvFTD** | **svPPA** | **nfvPPA** | **lvPPA** | **AD** | **PPD** | **Presymptomatic** | **Controls** | ***p*** | **Statistical difference** |
| --- | --- | --- | --- | --- | --- | --- | --- | --- | --- | --- |
| **NPI** | | | | | | | | | | |
| Delusions | 0.6 (2.0) | 0.9 (2.1) | 0.1 (0.24) | 0.4 (1.46) | 0.7 (1.9) | 2.1 (3.5) | 0.0 (0.1) | 0.1 (0.3) | <0.001 | pre = con = bvFTD = nfvPPA = lvPPA < PPD |
| Hallucinations | 0.7 (2.0) | 0.4 (1.9) | 0.0 (0.0) | 0.0 (0.0) | 0.2 (0.9) | 1.1 (2.9) | 0.0 (0.0) | 0.0 (0.3) | 0.001 | pre < PPD |
| Agitation/aggression | 1.9 (2.3) | 1.4 (2.7) | 0.7 (2.1) | 1.2 (2.5) | 0.6 (1.3) | 3.9 (2.1) | 0.1 (0.3) | 0.0 (0.2) | <0.001 | pre = con = AD < bvFTD = nfvPPA  = svPPA = lvPPA < PDD |
| Depression/dysphoria | 2.4 (3.8) | 2.5 (3.5) | 1.0 (2.1) | 1.1 (2.1) | 1.6 (2.2) | 4.9 (4.7) | 0.2 (0.8) | 0.3 (0.9) | <0.001 | con = pre < svPPA = AD = bvFTD  = nfvPPA = lvPPA < PPD |
| Anxiety | 1.8 (3.0) | 0.4 (0.9) | 1.0 (2.1) | 0.7 (1.4) | 1.4 (2.5) | 3.3 (4.3) | 0.0 (0.3) | 0.0 (0.1) | <0.001 | con = pre < AD = bvFTD = nfvPPA  = svPPA = lvPPA < PPD |
| Euphoria/elation | 0.7 (1.5) | 0.2 (0.7) | 0.3 (1.0) | 0.2 (1.0) | 0.1 (0.4) | 0.6 (1.7) | 0.0 (0.1) | 0.0 (0.2) | 0.010 | con = pre < bvFTD |
| Apathy/indifference | 4.6 (3.9) | 2.8 (3.0) | 2.1 (3.5) | 1.2 (2.1) | 1.3 (1.8) | 4.4 (4.3) | 0.1 (0.2) | 0.1 (0.6) | <0.001 | con = pre = AD = lvPPA = svPPA  = lvPPA < PDD < bvFTD |
| Disinhibition | 4.0 (4.3) | 1.7 (3.4) | 0.3 (0.7) | 1.3 (3.1) | 0.6 (1.7) | 3.3 (3.1) | 0.1 (0.8) | 0.0 (0.1) | <0.001 | con = pre = AD = nfvPPA = svPPA  = lvPPA < PPD = bvFTD |
| Irritability/lability | 3.1 (3.8) | 1.1 (2.4) | 0.8 (1.7) | 0.9 (1.6) | 1.1 (2.2) | 5.2 (3.5) | 0.2 (0.7) | 0.2 (0.9) | <0.001 | con = pre = nfvPPA = svPPA = lvPPA  = AD < bvFTD < PPD |
| Aberrant motor behaviour | 2.4 (3.7) | 0.5 (1.3) | 0.4 (1.2) | 0.7 (2.0) | 0.6 (1.6) | 3.0 (3.9) | 0.0 (0.1) | 0.1 (0.5) | <0.001 | con = pre = nfvPPA = svPPA = lvPPA  = AD < bvFTD = PPD |
| Night-time behaviour | 2.3 (3.6) | 1.4 (3.5) | 1.3 (3.3) | 0.7 (1.5) | 0.7 (1.7) | 6.1 (4.7) | 0.2 (1.0) | 0.6 (2.2) | <0.001 | con = pre < bvFTD = AD = nfvPPA  = svPPA = lvPPA < PDD |
| Appetite/Eating | 5.8 (4.5) | 3.2 (5.0) | 1.1 (1.9) | 1.7 (3.1) | 1.4 (2.6) | 3.0 (3.7) | 0.1 (0.3) | 0.1 (0.6) | <0.001 | con = pre < AD = nfvPPA = svPPA  = lvPPA = PDD < bvFTD |
| **FTD Module** | | | | | | | | | | |
| Loss of sympathy/empathy | 6.4 (4.6) | 3.8 (4.5) | 1.1 (1.8) | 2.1 (3.9) | 1.2 (2.4) | 4.3 (4.8) | 0.3 (1.0) | 0.1 (0.8) | <0.001 | con = pre = AD = nfvPPA =  svPPA = lvPPA < PDD = bvFTD |
| Ritualistic/compulsive behaviour | 3.6 (4.2) | 1.7 (2.5) | 1.1 (2.4) | 1.8 (4.0) | 1.8 (3.2) | 2.9 (4.7) | 0.0 (0.0) | 0.2 (1.2) | <0.001 | con = pre < bvFTD = PDD |
| Poor response to social/emotional cues | 6.5 (4.4) | 2.9 (3.5) | 1.7 (2.3) | 1.8 (4.0) | 1.4 (2.6) | 3.9 (4.3) | 0.1 (0.7) | 0.0 (0.2) | <0.001 | con = pre = AD = nfvPPA = svPPA  = lvPPA < PDD < bvFTD |
| Inappropriate trusting behaviour | 2.4 (3.4) | 1.5 (3.5) | 0.5 (1.1) | 0.4 (1.0) | 0.5 (1.7) | 0.8 (2.2) | 0.0 (0.1) | 0.1 (0.4) | <0.001 | con = pre = AD < PDD = bvFTD |
| Hyperreligiosity | 0.7 (2.5) | 0.0 (0.0) | 0.0 (0.0) | 0.0 (0.0) | 0.0 (0.0) | 0.7 (2.3) | 0.0 (0.0) | 0.0 (0.0) | 0.005 | - |
| Hypersexuality | 0.8 (2.4) | 0.2 (0.7) | 0.1 (0.2) | 0.7 (2.9) | 0.0 (0.2) | 2.6 (4.5) | 0.0 (0.0) | 0.0 (0.0) | <0.001 | con = pre = AD = bvFTD = nfvPPA  = svPPA = lvPPA < PDD |
| Altered sense of humour | 3.0 (3.8) | 1.6 (2.7) | 0.1 (0.2) | 0.6 (2.0) | 0.3 (0.9) | 2.2 (2.9) | 0.1 (0.3) | 0.0 (0.0) | <0.001 | con = pre = AD = nfvPPA = lvPPA  < svPPA = PDD = bvFTD |
| Altered responsiveness to pain and/or temperature | 3.0 (4.0) | 1.6 (2.5) | 0.4 (1.5) | 1.1 (2.1) | 1.4 (2.6) | 2.8 (3.7) | 0.0 (0.0) | 0.0 (0.0) | <0.001 | con = pre = AD = nfvPPA = lvPPA  < svPPA = PDD = bvFTD |
| **Total NPI** | 30.5 (21.4) | 17.7 (20.0) | 4.7 (8.3) | 10.0 (14.0) | 9.8 (11.6) | 40.8 (28.6) | 1.0 (2.4) | 1.7 (4.7) | <0.001 | con = pre = AD = nfvPPA = lvPPA  < svPPA = PDD = bvFTD |
| **Total NPI with FTD Module** | 55.9 (37.8) | 32.4 (33.4) | 8.8 (11.7) | 15.1 (28.4) | 16.6 (18.8) | 61.1 (43.6) | 1.5 (4.1) | 2.1 (6.3) | <0.001 | con = pre = AD = nfvPPA = lvPPA  < svPPA = PDD = bvFTD |
| Values indicate: mean (standard deviation). Abbreviations: NPI, neuropsychiatric inventory; FTD, frontotemporal dementia; bvFTD, behavioural variant frontotemporal dementia; svPPA, semantic variant primary progressive aphasia; nfvPPA, non-fluent variant primary progressive aphasia; lvPPA, logopenic variant primary progressive aphasia; AD, Alzheimer’s dementia; PPD, primary psychiatric disorder; pre, presymptomatic mutation carrier; con, controls. | | | | | | | | | | |
